# OLDER ADULTS’ PERCEPTIONS OF HOPE REGARDING THEIR ABILITY TO MAKE DIET AND EXERCISE CHANGES

**DOI:** 10.1093/geroni/igad104.3504

**Published:** 2023-12-21

**Authors:** Amber Worthington, Dale Golden, Britteny Howell

**Affiliations:** University of Alaska Anchorage, Anchorage, Alaska, United States; University of Alaska Anchorage, Anchorage, Alaska, United States; University of Alaska Anchorage, Anchorage, Alaska, United States

## Abstract

Successful aging is facilitated by healthy diet and exercise levels; however, older adults may face barriers to obtaining these. One key motivator for improving older adults’ diet and exercise may be perceptions of hope. Persuasive Hope Theory (PHT) posits that hope motivates behavior by focusing peoples’ thoughts on opportunities for actions that lead to future rewards. Research suggests hope is particularly motivational for older adults, yet little work has examined factors that lead older adults to feel hope. In line with PHT, this project examined whether older adults’ perceptions that increasing fruit and vegetable intake (FVI) and exercise are important, possible, congruent with their goals (i.e. goal congruence), and will lead to a better future (i.e., future expectation), influence feelings of hope. A convenience sample of participants (N=58) aged 57-87 (M=71.98, SD =8.74) living in urban Alaska completed a questionnaire as part of a larger study. A multiple regression analysis revealed that importance (M=4.40, SD=0.67) and future expectation (M=4.47, SD=0.66) for increasing FVI significantly predicted feeling hopeful about increasing FVI (M=4.40, SD=0.75), but perceptions that increasing FVI is possible (M=4.26, SD=0.74) and goal congruent (M=4.34, SD=0.64) did not (F(4, 53)=11.29, p<.05, R2=.46). The PHT variables for increasing exercise did not significantly predict feeling hopeful about increasing exercise. These results suggest PHT, specifically importance and future expectation, may be used to strategically design messages to increase older adults’ hopeful feelings for increasing FVI; however, more research is needed due to the limitations of the small convenience sample.

